# DECONbench: a benchmarking platform dedicated to deconvolution methods for tumor heterogeneity quantification

**DOI:** 10.1186/s12859-021-04381-4

**Published:** 2021-10-02

**Authors:** Clémentine Decamps, Alexis Arnaud, Florent Petitprez, Mira Ayadi, Aurélia Baurès, Lucile Armenoult, HADACA consortium, N. Alcala, A. Arnaud, F. Avila Cobos, Luciana Batista, A.-F. Batto, Y. Blum, F. Chuffart, J. Cros, C. Decamps, L. Dirian, D. Doncevic, G. Durif, S. Y. Bahena Hernandez, M. Jakobi, R. Jardillier, M. Jeanmougin, P. Jedynak, B. Jumentier, A. Kakoichankava, Maria Kondili, J. Liu, T. Maie, J. Marécaille, J. Merlevede, M. Meylan, P. Nazarov, K. Newar, K. Nyrén, F. Petitprez, C. Novella Rausell, M. Richard, M. Scherer, N. Sompairac, K. Waury, T. Xie, M.-A. Zacharouli, Sergio Escalera, Isabelle Guyon, Rémy Nicolle, Richard Tomasini, Aurélien de Reyniès, Jérôme Cros, Yuna Blum, Magali Richard

**Affiliations:** 1 Laboratory TIMC-IMAG, UMR 5525, CNRS, Univ. Grenoble Alpes, Grenoble, France; 2 Data Institute, Univ. Grenoble Alpes, Grenoble, France; 3 Programme Cartes d’Identité des Tumeurs (CIT), Ligue Nationale Contre le Cancer, Paris, France; 4 Universitat de Barcelona and Computer Vision Center, Barcelona, Spain; 5 LISN (INRIA/CNRS), Université Paris-Saclay, Gif-sur-Yvette, France; 6 INSERM U1068 CRCM, Marseille, France; 7 Dpt of Pathology, Beaujon Hospital, Univ. Paris-INSERM U1149, Clichy, France; 8 IGDR UMR 6290, CNRS, Université de Rennes 1, Rennes, France

**Keywords:** Benchmarking platform, Deconvolution, Transcriptome, DNA methylation, Omics integration, Cellular heterogeneity, Cancer

## Abstract

**Background:**

Quantification of tumor heterogeneity is essential to better understand cancer progression and to adapt therapeutic treatments to patient specificities. Bioinformatic tools to assess the different cell populations from single-omic datasets as bulk transcriptome or methylome samples have been recently developed, including reference-based and reference-free methods. Improved methods using multi-omic datasets are yet to be developed in the future and the community would need systematic tools to perform a comparative evaluation of these algorithms on controlled data.

**Results:**

We present DECONbench, a standardized unbiased benchmarking resource, applied to the evaluation of computational methods quantifying cell-type heterogeneity in cancer. DECONbench includes gold standard simulated benchmark datasets, consisting of transcriptome and methylome profiles mimicking pancreatic adenocarcinoma molecular heterogeneity, and a set of baseline deconvolution methods (reference-free algorithms inferring cell-type proportions). DECONbench performs a systematic performance evaluation of each new methodological contribution and provides the possibility to publicly share source code and scoring.

**Conclusion:**

DECONbench allows continuous submission of new methods in a user-friendly fashion, each novel contribution being automatically compared to the reference baseline methods, which enables crowdsourced benchmarking. DECONbench is designed to serve as a reference platform for the benchmarking of deconvolution methods in the evaluation of cancer heterogeneity. We believe it will contribute to leverage the benchmarking practices in the biomedical and life science communities. DECONbench is hosted on the open source Codalab competition platform. It is freely available at: https://competitions.codalab.org/competitions/27453.

**Supplementary Information:**

The online version contains supplementary material available at 10.1186/s12859-021-04381-4.

## Background

The recent development of high-throughput sequencing technologies has enabled the characterization of the genetic regulations underlying diseases such as cancer. Important advances have been made but studies often overlook the fact that tumors are made up of cells from different identities and origins. The quantification of tumor heterogeneity is of great interest to the biomedical research community because the various components of a tumor are key factors in tumor progression, clinical outcome and response to therapy. To isolate a cell population of interest, microdissection techniques can be performed on clinically heterogeneous tissue samples, but these advanced techniques are not feasible in clinical routine. In addition, single-cell technologies, while promising, have intensive protocols and require expensive and specialized resources, currently hindering their establishment in a clinical setting [1]. Instead, deconvolution methods can be used to infer cell-type composition in silico from bulk measurements, which enable the analysis of a large number of publicly available omic datasets. Bioinformatics tools that assess the different cell populations from bulk transcriptome [2–5] and methylome [6–9] samples have been recently developed, including reference-based and reference-free methods.

Recent efforts have been made to objectively compare existing tools in order to guide the users. In particular, two recent benchmark studies proposed a comprehensive comparison of transcriptome-based deconvolution methods using various parameters and simulation settings [10, 11]. In the same vein, the DREAM challenge proposed in 2019 [12] a data challenge dedicated to the prediction of immune cell types, showing the emerging spirit towards reproducibility and benchmarking. Although interesting, all these efforts are time-bound and cannot take into account upcoming novel methods. Moreover, the possibility to integrate different types of omic data to infer cell-type proportions is currently under-studied.

Standardized unbiased benchmarking resources are essential to evaluate the performances of computational methods. Indeed, these resources should avoid falling into the ‘self-assessment trap’, in which researchers are unrealistically expected to fairly compare their own computational method with other similar algorithms [13, 14]. In addition, unbiased attempts to benchmark computational methods are often static in space and time, preventing further contributions of other scientists or the assessment of new methods developed after the publication of the benchmark [15]. Recent collective initiatives provided formal guidelines and unified frameworks to improve unbiased performance evaluation [16]. For instance, the Global Alliance for Genomic and Health (GA4GH) published an open access benchmarking tool to assess germline small variant calls in human genomes [17]. More recently, BEELINE, a uniform interface to evaluate Gene Regulatory Network inference from single-cell data, was published and made freely accessible in the form of a docker image [18].

In this project, we built on a previous HADACA (Health Data Challenge consortium) benchmarking study [7] to develop a standardized benchmark framework for accurately evaluating quantification of tumor intra-heterogeneity from a multi-omic dataset. First, we built in silico 10 paired methylome and transcriptome benchmark datasets, using pancreatic cancer (PDAC, pancreatic adenocarcinoma) as a case study. These benchmark datasets were made realistic by the integration of the latest knowledge on PDAC biology [19–21] in the simulation models and can be used as ‘truth’ to evaluate computational methods quantifying tumor heterogeneity. Second, we defined Mean Absolute Error (MAE) on estimated cell-type proportions and computational time as standard performance metrics. Third, we embedded the benchmark dataset and the scoring algorithm into a web platform called DECONbench. This web platform enables continuous and crowdsourced benchmarking, by asking participants to submit source code of their algorithm. Each submission is therefore run by the platform on the benchmark dataset and results generated in a reproducible way. Fourth, we implemented on the platform baseline methods based on some previously published deconvolution algorithms and tools. Therefore, DECONbench is an open resource to evaluate novel computational methods in an unbiased way. It provides a private general report on the overall performances of the method submitted by any participant and offers the possibility to share all source code of the contributing methods, as well as performance evaluation on a public leaderboard.

Here we present DECONbench, an innovative public benchmarking platform, open source and freely available, aiming at comparing integrative deconvolution methods for tumor heterogeneity quantification. This framework supports both crowdsourcing benchmarking (collaborative and competitive assessment of the methods) and continuous benchmarking (possibility to continuously integrate novel methods), two features that should contribute to the widespread community adoption of benchmarking good practices [15, 22]. To conclude, DECONbench is an open online benchmark framework including gold standard benchmarking datasets from different types of omic data, state-of-the-art baseline computational methods and it enables the submission of new methods for evaluation.

## Implementation

### The benchmarking platform infrastructure

**Fig. 1:**
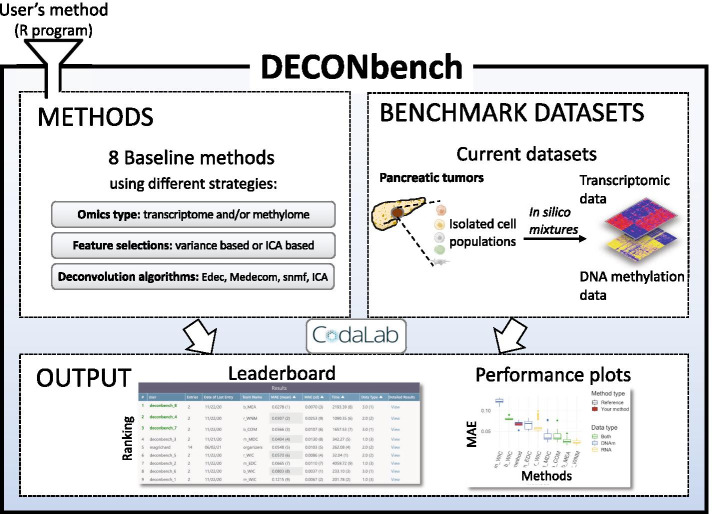
Overview of the DECONbench platform. The platform proposes a set of 8 baseline deconvolution methods and benchmark datasets consisting of paired methylome and transcriptome of in silico mixtures from pancreatic tumors. The platform releases the performance of each method on a leaderboard and provides plots for deeper evaluation. New methods are automatically compared to the existing ones

DECONbench takes advantage of the Codalab web-based platform (https://competitions.codalab.org/) to provide a software environment for evaluating deconvolution methods. Users submit a full program that is applied to the provided benchmark datasets and compared to the ground truth. DECONbench outputs a performance score displayed on the leaderboard (Fig. 1).

### Usage

DECONbench is optimized to execute methods developed in R statistical programming language, using a docker image provided on our website. The benchmark is structured around an ingestion program used as a wrapper object to execute an R program. Should anyone wish to benchmark a method coded in another language, R could then be used as a script language to execute the given program by invoking a System Command. A list of R packages installed on the docker image is as well provided. Users need: (i) to register to DECONbench on the participate tab and to download the starting kit and the public datasets; (ii) to develop an algorithm according to DECONbench guidelines; (iii) to submit their code (as a zip file) in the participate tab. Submitted algorithms are evaluated on DECONbench datasets and benchmarked with the other baseline methods. Users should note that methods relying on stochastic algorithms will give slightly variable performance on each run, unless an initialization is specified in the source code. Resulting scores appear on the leaderboard and a fact sheet is edited summarizing the performances. Importantly, users can choose whether they want their algorithm to be public or private.

## Results

### Provided benchmark datasets

**Fig. 2:**
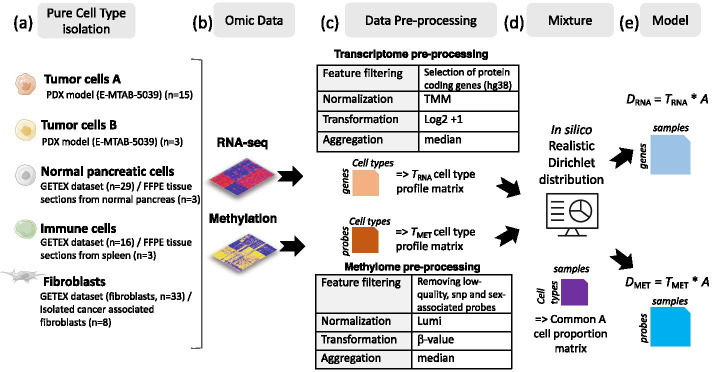
Benchmark dataset construction: **a** 5 different cell populations present in pancreatic tumors were considered. **b** Raw transcriptome and methylome profiles of these different cell populations were extracted from various sources (PDX model, tissues or isolated cells). **c** Raw cell type profile matrices were preprocessed together (Feature filtering, normalization, signal transformation, sample aggregation) to avoid any batch effect. After pre-processing, transcriptomic data are constituted of log2-transformed expression counts on 21,566 genes and methylome data of beta-values on 772,316 EPIC array CpG sites. **d** In silico Dirichlet distributions have been used based on realistic proportions defined by the anatomopathologist expertise (J. Cros). **e** Paired methylome and transcriptome of in silico mixtures from pancreatic tumors were obtained by considering *D* = *T* × *A*, with T the cell-type profiles (matrix of size *M* × *K*, with M the number of features and K = 5 the number of cell types) and A the cell-type proportion per patient (matrix of size *K* × *N,* with *N* = *30* the number of samples) common between both omics. One training set (*D*_*MET*_* and D*_*RNA*_) is accessible to the users (obtained by one realization of A). The algorithms are compared on 10 test sets (obtained from 10 other realizations of A) that are hidden on the platform

We have generated paired transcriptome and methylome benchmarking datasets from primary cells from pancreatic tumors and sorted cells from public datasets (Fig. 2). Gold standard heterogeneous samples were simulated using mixtures of individual cell populations (fibroblast, immune cells, normal epithelial cells and cancer cells, see “Methods” section). Exact sample compositions are not accessible to the users. Participants are facing a deconvolution problem to solve the following model: $D=TA$, with $D$ the complex matrix of molecular profiles measured on heterogeneous samples; $T$, a reference matrix of cell-type specific molecular profiles; and $A$, a proportion matrix of cell-type abundance in each sample. The aim of the competition is to find the best estimate of the proportion matrix $A$. Methods are evaluated on their accuracy to estimate the cell-type proportions per sample from transcriptome and/or methylome heterogeneous profiles. The discriminating metric is the mean absolute error (MAE, see “Methods” section) between the estimate and the ground truth.

### Selection of baseline methods from a data challenge

We used these unreleased benchmark datasets in a data challenge aiming at inferring cell-type proportions from a cancer dataset including both transcriptome and methylome profiles (https://tinyurl.com/hadaca2019). Baseline methods provided on DECONbench were collectively designed, tested and implemented during the challenge. They are composed of two steps: first, we operate a feature selection process to reduce the dimensions of the dataset, second, we apply a deconvolution algorithm. These algorithms consist of various statistical tools already published, based on unsupervised source separation approaches: ICA-based (Independent Component Analysis) [23–25] or NMF-based (Non-negative Matrix Factorization) [8, 9, 26]. Each baseline method was designed to be applied either on single-omic (see Table 1, Data type “RNA” or “DNAm”) or in an integrated fashion on both the transcriptome and the methylome dataset (see Table 1, Data type “both” and Multi-omic integration strategy). As baseline on DECONbench, we implemented the eight methods that predict the real cell proportions with the highest accuracy (i.e. lowest MAE between the estimate and the ground truth) (Table 1). All baseline methods source code are publicly accessible on the platform.

**Table 1:** Description of each baseline method included in the benchmark

Name	RNA_wICA	RNA_wNMF	DNAm_EDec	DNAm_MeDeCom	DNAm_wICA	both_wICA	both_wNMFMeDeCom	both_meanwNMFMeDeCom
Acronym	**r_WIC**	**r_WNM**	**m_EDC**	**m_MDC**	**m_WIC**	**b_WIC**	**b_COM**	**b_MEA**
Data type	RNA	RNA	DNAm	DNAm	DNAm	both	both	both
Feature Selection DNAm	/	/	5.000 most variable probes	5.000 most variable probes	5.000 most variable probes	5.000 most variable probes	5.000 most variable probes	5.000 most variable probes
Feature Selection RNA	ICA, selection of top-contributing genes and filtering of duplicated genes	ICA, selection of top-contributing genes	/	/	/	/	ICA, selection of top-contributing genes	ICA, selection of top-contributing genes
Deconvolution algorithm DNAm	/	/	Edec	MeDeCom	ICA weighted by top-contributing probes	ICA weighted by top-contributing probes	MeDeCom with the A matrix computed on RNA as startA parameter	MeDeCom
Deconvolution algorithm RNA	ICA weighted by top-contributing genes	NMF with snmf/r method	/	/	/	ICA weighted by top-contributing genes	NMF with snmf/r method	NMF with snmf/r method
Multi-omic integration strategy	/	/	/	/	/	Averaged DNAm and RNAm proportion matrix	DNAm deconvolution uses RNA deconvolution as input	Averaged DNAm and RNAm proportion matrix
Time 10 A	~ 10 min	~ 20 min	~ 3 h	~ 17 h	~ 10 min	~ 10 min	~ 17 h	~ 17 h 30 min
Time 1 A	~ 1 min	~ 2 min	~ 20 min	~ 1 h 40	~ 1 min	~ 1 min	~ 1 h 40 min	~ 1 h 45 min
Reference of the tools/algorithms used	Hyvarinen [25]	Frichot et al. [26]	Onuchic et al. [9]	Lutsik et al. [8]	Hyvarinen [25]	Hyvarinen [25]	Lutsik et al. [8] and Frichot et al. [26]	Lutsik et al. [8] and Frichot et al. [26]

A baseline method is composed of two steps: [1] feature selection and [2] deconvolution algorithm. All deconvolution algorithms used as baseline are already published and documented in the literature (see Reference of the tools/algorithms used). A detailed description of the coding instruction and a mathematical description of the algorithms can be found in the "Methods" section. Source code is publicly available on the DECONbench platform. Time 10 A corresponds to the approximated computation time to estimate 10 proportion matrices A (corresponding to the test sets hidden on the platform). Time 1 A corresponds to the approximated computation time to estimate 1 proportion matrix A (closer to real applications on one dataset)

**Fig. 3:**
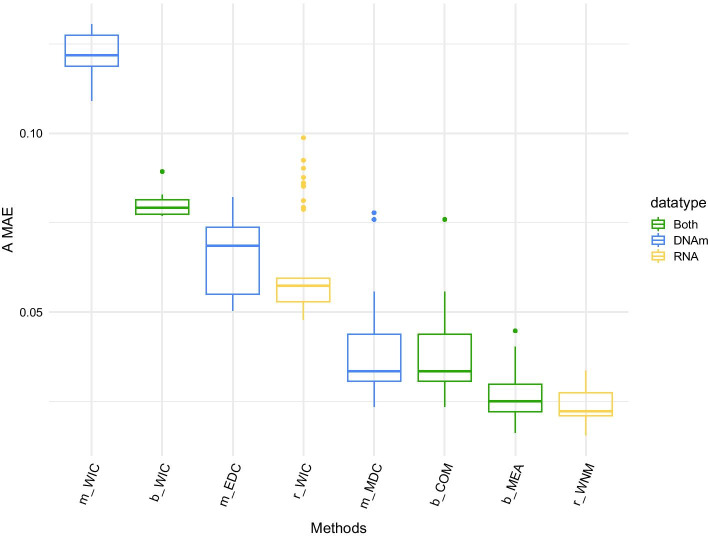
Baseline algorithm performances: Boxplots of ‘MAE A’ obtained for reference algorithms (‘MAE A’: Mean Absolute Error on estimated A, the matrix of cell proportion per patient). Each reference algorithm was run 5 times on each simulated data, for 10 independent simulations (50 measures were used to construct the boxplots). Each color represents the type of data used to infer A (‘DNAm’ for *D*_*MET*_ training set, ‘RNA’ for *D*_*MET*_ training set and ‘Both’ for methods integrating *D*_*MET*_ and *D*_*RNA*_). Details of the algorithms can be found in the "Methods" section

**Fig. 4:**
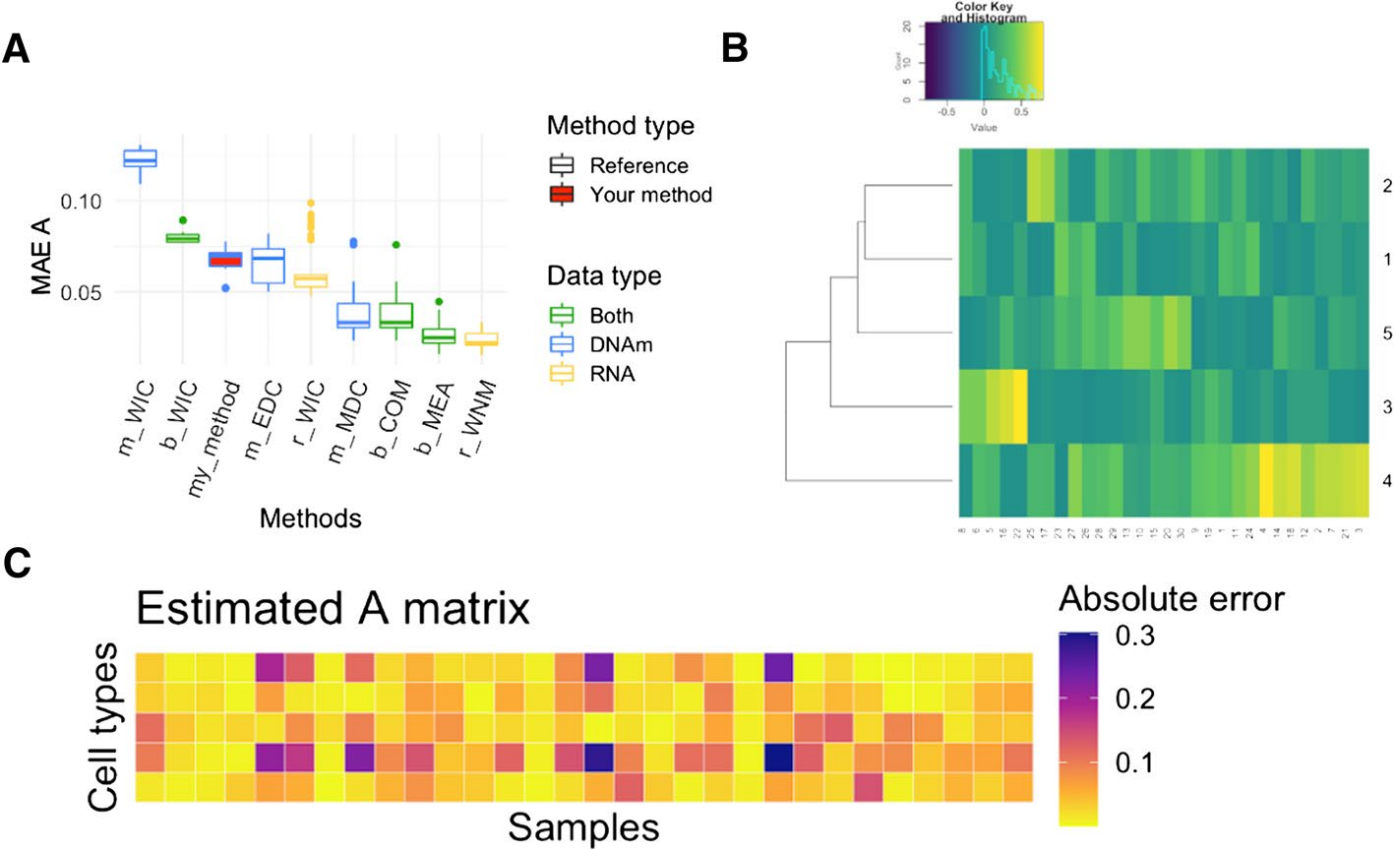
DECONbench graphical outputs. **a** Boxplots of the Mean Absolute Errors (MAE) of the estimations of the A matrices (i.e. proportion matrices) obtained for each method that uses the transcriptome only (yellow), the methylome only (blue) or both omics (green). Boxplots of the baseline methods and other existing methods are shown in white, whereas the user's method is shown in red. **b** Heatmap of each A-matrix estimate are generated. The cell populations are in rows and the samples in columns. **c** Heatmaps of Absolute Error of each proportion estimate are generated. The cell populations are in rows, and the samples in columns

Bold acrononyms are used to identify methods in Figs. 3 and 4

### Performance of the baseline single-omic methods

We run all the baseline methods on 10 different simulated datasets and computed the corresponding MAEs (Fig. 3). The best algorithms based on single-omic datasets were the r_WNM method for RNA-based data (mean MAE of 0.024) and the m_MDC method for DNAm-based data (mean MAE of 0.038). Both are NMF-based algorithms, details on the methods can be found in the "Methods" section. DECONbench provides also the computing time for each method, as an indicator of algorithms optimization. It is worth underlying that the computation time of m_MDC algorithm is significantly higher than the other DNAm-based methods we explored, suggesting that even high performance single-omic algorithms might be further optimized.

### Performance of the integrative multi-omics methods

Next, we tested basic multi-omic approaches averaging the results of single-omic methods: (i) the b_WIC method averages the proportion matrices given by the independent applications of independent component analysis (ICA) based deconvolution approach to transcriptome and methylome data, (ii) the b_MEA method computes an average proportion matrix from the output of the two best single-omic methods r_WNM and m_MDC. Averaging the ICA based approaches (b_WIC) gave intermediate performances (multi-omic accuracy equivalent to the mean of single-omic accuracies). Similarly, we did not observe increased performances when averaging the predicted proportion matrices of the two best methods (b_MEA).

We also proposed an integrative method (b_COM) based on transcriptome and methylome data. The best performing methylome-based method (m_MDC) relies on the MeDeCom tool which is a NMF-based deconvolution algorithm that performs multiple random initializations of the cell-type proportion matrix. Instead of using random initialization, we initialized the MeDeCom algorithm with the proportion matrix obtained from a NMF-based deconvolution of transcriptome data. Surprisingly, we did not observe a substantial performance improvement when integrating RNA deconvolution output into DNAm deconvolution algorithm (b_COM method, resulted in an average error decrease of 2.12% compared to m_MDC). These results highlight the need to further develop new methods to improve integration of multi-omic deconvolution algorithms.

### Toward crowdsourced and continuous benchmarking

As an example of continuous benchmarking, we used DECONbench to assess the performances of two recently evaluated single-omic algorithms in a comprehensive benchmark of reference-based deconvolution pipelines. We selected the Ordinary Least Square (OLS) and Robust Linear Regression (RLR) approaches, which have been shown to be effective in estimating cellular composition of simulated bulk healthy pancreatic transcriptomes [10]. We implemented the methods as recommended by Avila Cobos et al., including the generation of cell-type reference profiles from a pancreatic single-cell dataset [27] (see supplemental information for source code: Additional file 1: Source code). Interestingly, the performance of these methods is not better than the baseline methods, possibly due to the use of healthy pancreatic cells as a reference to estimate the composition of a simulated pancreatic adenocarcinoma (Additional file 1: Figure S1). These results suggest that further optimization should be considered to properly assess the performance of the OLS and RLR methods. This crowdsourced and continuous integration is now made possible thanks to our DECONbench platform.

## Conclusion

The DECONBench platform is a unique opportunity to compare the performance of deconvolution methods on different omics data. It can be used to assess the performance of newly developed methods by applying them on high quality benchmark datasets in a user-friendly fashion. Currently, the accuracy of new methods can be compared with the eight baseline methods that have been included in the benchmarking platform. As compared with previous time-bound comprehensive benchmarks of deconvolution methods (see Avila Cobos et al. [10]), our platform provides the possibility to continuously test and integrate newly developed methods, rather than focusing on an exhaustive comparison of existing tools. The baseline methods and user’s methods performances are reported on the leaderboard and on the graphical output of DECONBench (Fig. 4). The source code of the baseline methods can be downloaded directly on the DECONbench platform. The structure of DECONbench is open to evolution. Work is ongoing to generate new benchmark datasets including other omic types that will be added to the platform. In the near future, we plan to expand the usability of DECONbench by offering the possibility for owners of benchmark datasets to directly upload them on the platform.

DECONbench evaluation framework presents standard benchmark limitations [15, 16], such as the use of artificial in silico simulated data that do not capture the real experimental complexity, or the ranking of the methods based on a single performance metric. We would like to emphasize that MAE as scoring metric is only an imperfect proxy to evaluate quantification of tumor heterogeneity, as it does neither reflect the accuracy of cell-type specific molecular profile prediction (i.e. biological significance of inferred components), nor the correlation of estimated heterogeneity with real clinical outputs (such as prognosis or survival).

Overall, our platform will guide computational biologists to use the best proposed deconvolution algorithms and allow health professionals and biologists to obtain more accurate information regarding the composition of their samples, an important step towards personalized healthcare.

## Methods

### Data collection and preprocessing

For both transcriptome and methylome in silico mixtures, the same five cell types present in pancreatic tumors were considered (Fig. 2a, b): tumor cells A, tumor cells B, normal pancreatic cells, immune cells and fibroblasts. Pure cell type transcriptome profiles were retrieved from the GTEX RNA-seq dataset for the immune and normal pancreatic cell types (https://gtexportal.org/) and a previously published pancreatic tumor patient derived xenograft (PDX) RNA-seq dataset (E-MTAB-5039) for the two tumor cell types (total of 96 pure transcriptome profiles with 3 to 33 replicates per cell type) (Fig. 2c). Pure cell type methylation profiles were retrieved from the same samples of the PDX dataset for the two tumor cell types and tissue or isolated cell profiles were used for the microenvironment cell types (total of 32 methylome pure profiles with 3 to 15 replicates per cell type). Transcriptome dataset was restricted to protein coding genes and subjected to TMM normalization using the edgeR R package and log2 transformation. For the methylation data, we used the beta-value DNA methylation scores and removed probes with low-quality, that contained SNPs or located on sex chromosomes. Data were then adjusted for color balance bias and normalized between samples using the SSN (shift and scaling normalization) method using the lumi package functions (Fig. 2c). For both omics, the median of the replicate profiles for each cell type was calculated to compute the *T*_RNA_ and *T*_*MET*_ matrices, representing the cell type specific profiles for each omic. The median calculation may prevent underlying germline differences. These matrices were used for the in-silico mixtures, as detailed in the next sections (Fig. 2d, e).

### Formulation of the deconvolution problem

When a sample is constituted of K cell types, we assume that the level of methylation or gene expression observed in a bulk measurement of this biological sample (containing different cell types) results from a linear mixture of the K cell-type specific molecular profile weighted by the true cell-types proportions present in the sample. This assumption leads to the following models:

1 $$D_{MET} = T_{MET} A$$

2 $$D_{RNA} = T_{RNA} A$$

where *D*_*MET*_ is a (M × N) methylation matrix from ${\rm N}$ bulk heterogeneous samples with ${D}_{{MET}_{\{m,n\}}}$ the measured measured methylation (beta-value) of the m*th* CpG site for the n*th* sample representing the measured methylation (beta-values) for ${\rm N}$ samples; *D*_RNA_ is a (G × N) gene expression matrix from the same N bulk heterogeneous samples with ${D}_{{RNA}_{\{g,n\}}}$ the measured gene expression (normalized pseudo-log counts) of the g*th* gene for the n*th* sample; *T*_*MET*_ is an unknown (M × K) reference-profile matrix with ${T}_{{MET}_{\{m,k\}}}$ representing the average methylation beta-value of CpG site $m$ for the cell-type $k$; *T*_RNA_ is an unknown (G × K) reference-profile matrix with ${T}_{{RNA}_{\{g,k\}}}$ representing the average expression value (normalized pseudo-log counts) of gene $g$ for the cell-type $k$; and $A$ a (K × N) matrix representing the cell-type composition of the $N$ heterogeneous samples for $K$ cell types (i.e. the cell-type proportions), with ${A}_{\{k,n\}}$ the proportion of the n*th* sample for the k*th* cell type. Specifically, the $A$ proportion matrix is shared between the two models, as *D*_*MET*_ and *D*_*RNA*_ bulk molecular profiles are measured on the same biological samples. In the methods tested, $A$ is estimated with the following constrain: $\sum_{k=1}^{K}{A}_{kn}=1$ .

### Data modeling

The benchmark simulated bulk molecular profiles are constituted of 10 paired *D*_*MET*_ and *D*_*RNA*_ matrices. Simulations are processed as follows:

#### Step 1: Simulation of the shared proportion matrices

The mixture proportions of the matrices A were sampled from a Dirichlet distribution based on realistic biological composition of a pancreatic tumor, with the variation of Dirichlet parameters set to ${\alpha }_{0}=10$ for global cell composition (fibroblasts, immune, normal epithelial and cancer epithelial), and a variation of Dirichlet parameters set to ${\alpha }_{0}=1$ for cancer cells subpopulations (cancer basal-like and cancer classic). Exact proportion parameters are kept private to ensure unbiased evaluation of the methods.

#### Step 2: Simulation of the bulk D bulk matrices

We use the mathematical models (1) and (2) to simulate the bulk matrices, as previously described in Decamps et al. (2020) [7]. *D*_*MET*_ is a methylation matrix composed of 772,316 methylation values (EPIC array CpG sites) for N = 30 samples, D_MET_ was constructed as follows: *D*_*MET*_ = *T*_*MET*_* A*, with *T*_*MET*_ a matrix of K = 5 cell type-specific methylation reference profiles (methylation beta-values for each cell type considered: tumor cells A, tumor cells B, normal pancreatic cells, immune cells and fibroblasts), and *A* a (K $\times$ N) proportion matrix composed of K = 5 cell type proportions for each N = 30 sample. D_RNA_ is a transcriptome matrix composed of 21,566 gene expression values (normalized log-2 transformed RNA-seq counts values for each cell type) for N = 30 samples. D_RNA_ was constructed according to the following model: *D*_*RNA*_ = *T*_*RNA*_* A*, with *T*_*RNA*_ a matrix of the K = 5 cell type-specific transcriptome reference profiles (21,566 gene expression values for each cell type: tumor cells A, tumor cells B, normal pancreatic cells, immune cells and fibroblasts), and A the same (K $\times$ N) proportion matrix used to simulate D_MET_.

#### Step 3: Simulation of a technical noise

We added a generic Gaussian noise on each bulk simulated matrix using the following parameters: mu = 0 and sd = 0.05.

#### Step 4: Replication of the simulations

To ensure robustness of the method's evaluation, we generated 10 replications of paired D_MET_ and D_RNA_ matrices, using independent simulation of A proportions matrices. For each pair of D_MET_ and D_RNA_ matrices, the same T_MET_ and T_RNA_ reference matrices were used.

### Performance evaluation

The aim of deconvolution algorithms is to correctly estimate the proportion matrix A. We evaluated algorithm performances by computing the mean absolute error (MAE), as previously described in Decamps et al. (2020) [7]:

3 $$\mathrm{MAE}=\frac{{\sum }_{\mathrm{n}=1}^{\mathrm{N}}{\sum }_{\mathrm{k}=1}^{\mathrm{K}}|{\mathrm{Aest}}_{\mathrm{nk}}-{\mathrm{Areal}}_{\mathrm{nk}}|}{\mathrm{NK}}$$

One training set (*D*_*MET*_* and D*_*RNA*_) is publicly available (the *A, T*_*RNA*_ and *T*_*MET*_ matrices used for compute *D*_*MET*_* and D*_*RNA*_ matrices remain private, as they are directly involved in performance evaluation). The algorithms are evaluated on 10 test sets (*D*_*MET*_* and D*_*RNA*_), obtained from 10 independent realizations of A, given the simulation models *D*_*MET*_ = *T*_*MET*_* A and D*_*RNA*_ = *T*_*RNA*_* A*. These test sets are hidden on the platform to avoid overfitting. During evaluation of baseline algorithms, each algorithm was run 5 times on each simulated set of data, to account for randomness in algorithm outputs.

### Description of the baseline methods

The baseline methods we propose here are wrappers of already published unsupervised deconvolution algorithms (ICA-based or NMF-based). We assume here that A, *T*_*MET*_ and *T*_*RNA*_ are unknown and need to be estimated, either independently (single-omic pipelines) or integratively (double-omic pipelines). Before deconvolution, we systematically apply a pre-treatment step of dimensionality reduction based on feature selection. All baseline methods source code is downloadable on the DECONbench platform.

All baselines relies on unsupervised deconvolution algorithms, which consists in solving $D=TA$, either by ICA-based (i) or NMF-based (ii) approaches. (i) ICA-based approaches (r_WIC, m_WIC and b_WIC) consist of minimizing mutual information of sources by defining independent components. It is based on the fixed-point FastICA algorithm developed by Aapo Hyvärinen [24, 25]. (ii) NMF-based approaches (r_WNM, m_EDC, m_MDC, b_COM, b_MEA) aims to minimizing ${\left|\left|D-TA\right|\right|}_{2}$.

### RNA_wICA (r_WIC, ICA-based deconvolution on RNA)

The method RNA_wICA (r_WIC) uses transcriptomic data as input and is based on the ICA algorithm for both feature selection and deconvolution. It relies on the use of the functions “runICA” and “getGenesICA” developed by P. Nazarov (sablab.net/scripts/LibICA.r) and the deconica R package [23].

- *STEP1: feature selection* For the ICA-based feature selection, the function “runICA” is run at first with the parameters ncomp = 10 and ntry = 50. Then, the function “getGenesICA” selects top-contributing genes with a FDR of 0.2, the feature selection is done on these contributing genes belonging to a component having an average stability greater than 0.8. Finally, duplicated genes are removed.
- *STEP2: deconvolution* First, we perform FastICA unsupervised deconvolution *(*deconica::run_fastica is run with the parameters overdecompose = FALSE and n.comp = 5; remaining parameters are set to default). Second, we compute the abundance of the identified components, using the weighted-mean of the 30-top genes of each Independent Component (IC), in each sample as, a surrogate of the component signal. The 30 most important genes of each ICA component are extracted by the function deconica::generate_markers with the parameter return = "gene.ranked". These genes are used to weight the component scores in each patient (the weighted-score of a given IC in patient $p$ corresponds to the weighted mean expression of the 30-top genes on that component. We used, in the function deconica::get_scores, the log counts of the ICA as “df” parameter, the list of 30 genes as “markers.list” parameter, and the parameter summary = "weighted.mean". Finally, the estimated proportions are calculated from the inferred weighted-score with the function deconica::stacked_proportions_plot on the transpose of the deconica::get_scores output.

### DNAm_wICA (m_WIC, ICA-based deconvolution on DNAm)

The method DNAm_wICA (m_WIC) uses DNA methylation data as input.

- *STEP1: feature selection* It has no feature selection step.
- *STEP2: deconvolution* The deconvolution step is based on ICA, similarly to what was described for the second step of RNA_wICA, but applied on the DNA methylation matrix.

### both_wICA (b_WIC. ICA-based deconvolution on RNAD and DNAm)

The method both_wICA (b_WIC) combines transcriptomics and DNA methylation information.

- *STEP1: feature selection* It has no feature selection step.
- *STEP2: deconvolution* The deconvolution is in two steps, one on each data type. The transcriptomics and DNA methylation data are separately deconvoluted with the same deconvolution step as in r_WIC and m_WIC respectively to estimate A_MET_ and A_RNA_.
- *STEP3: integration* Finally, the mean of both A_MET_ and A_RNA_ estimated proportion matrices is computed as the final method output. To compute the average, the cell types of the both deconvolution matrices are matched by iteration. The cell types of the methylation result matrix are reordered 1000 times, and the one that best correlates with the transcriptomic result matrix is kept.

### RNA_wNMF (r_WNM, NMF-based deconvolution on RNA)

The method RNA_wNMF (r_WNM), is a two step-approach that uses transcriptomic data as input.

- *STEP1: feature selection* The first step uses ICA to perform a feature selection as described for RNA_wICA, although duplicated genes are kept. This step therefore allows genes that contribute to several components to be present several times in the data.
- *STEP2: deconvolution* The deconvolution is based on sparse NMF and least-squares optimization to minimize ${\left|\left|D-TA\right|\right|}_{2}$ [26]. It is called by the NMF::nmf function, with the parameter method = "snmf/r".

### DNAm_EDec (m_EDC, NMF-based deconvolution on DNAm)

- *STEP1: feature selection* The method DNAm_EDec (m_EDC), uses DNA methylation data as input and follows the pipeline implemented in the R package medepir [7]. The feature selection is performed by medepir::feature_selection for keeping highly variable probes (5000 most variable probes).
- *STEP2: deconvolution* The NMF-based algorithm of the method EDec [9] is used for the deconvolution part, with the function medepir::Edec and all the selected probes as “infloci” parameter. The algorithm consist in minimizing the error term ${\left|\left|D-TA\right|\right|}_{2}$ with constraints on methylation values: $0\le A\le 1$ and $0\le T\le 1$ and constraints on proportions ${\sum }_{k-1}^{K}{A}_{kn}=1$ where ${A}_{kn}$ is the proportion of the ${n}_{th}$ sample for the ${k}_{th}$ cell type.

### DNAm_MeDeCom (m_MDC, NMF-based deconvolution on DNAm)

- *STEP1: feature selection* The method DNAm_MeDeCom (m_MDC), uses DNA methylation data as input and is based on the pipeline of the R package medepir. The feature selection is performed as for DNAm_EDec above to select the 5000 most variable probes.
- *STEP2: deconvolution* The deconvolution step, however, uses the MeDeCom R package [8]. It is run with the function MeDeCom::runMeDeCom, with the lambda parameter set to 0.01. As EDec implementation of NMF algorithm, MeDeCom algorithm consists in minimizing the error term ${\left|\left|D-TA\right|\right|}_{2}$ with constraints on methylation values: $0\le A\le 1$ and $0\le T\le 1$ ; and constraints on proportions ${\sum }_{k-1}^{K}{A}_{kn}=1$ where ${A}_{kn}$ is the proportion of the ${n}_{th}$ sample for the ${k}_{th}$ cell type. It also uses a regularization function that favors methylation values close to 0 or 1.

### both_wNMFMeDeCom (b_COM, NMF-based deconvolution on RNA and DNAm)

The method both_wNMFMeDeCom (b_COM) combines transcriptomics and DNA methylation information. It is the combination of the two methods RNA_wNMF and DNAm_MeDeCom. The method r_WNM is first applied to the RNAseq matrix.

- *STEP1: feature selection* The DNA methylation matrix is pre-treated as described in the m_MDC method, with the selection of 5000 most variable probes.
- *STEP2-3: deconvolution-integration* Finally, the MeDeCom algorithm is run on the DNAm data, with the result of r_WNM as the initialization parameter startA.

### both_meanwNMFMeDeCom (b_MEA, NMF-based deconvolution on RNA and DNAm)

The method both_meanwNMFMeDeCom (b_MEA), which integrates transcriptomics and DNA methylation, applies r_WNM to the transcriptomics matrix, m_MDC to the DNA methylation matrix.

- *STEP1: feature selection* Feature selection is performed on D_MET_ and D_RNA_ matrices as described in r_WNM and m_MDC sections.
- *STEP2: deconvolution* Deconvolution is performed on D_MET_ and D_RNA_ matrices as described in r_WNM and m_MDC sections to estimate A_MET_ and A_RNA_ matrices.
- *STEP3: integration* We computed the mean of the two estimated A_MET_ and A_RNA_ matrices, similarly to b_WIC.

### Availability and requirements

- Project name: DECONbench
- Project home page: https://competitions.codalab.org/competitions/27453
- Operating system(s): Linux (CodaLab platform)/Debian (DECONbench)
- Programming language: Python (CodaLab platform)/R (DECONbench)
- Other requirements: none
- License: Apache 2.0 (CodaLab platform)/CeCILL (DECONbench)
- Any restrictions to use by non-academics: none

## Supplementary Information

**Additional file 1: Figure S1:** DECONbench benchmark of OLS and RLR methods: an example of graphical outputs of new contributions to the benchmark. **Source code**.

## Data Availability

DECONbench is hosted on the open source Codalab competition platform. It is freely available at: https://competitions.codalab.org/competitions/27453. Further documentation (online demo) is available at: https://deconbench.github.io/.

## Abbreviations

DNAm: DNA methylation
FDR: False discovery rate
ICA: Independent component analysis
MAE: Mean absolute error
NMF: Non-negative matrix factorization
sd: Standard deviation
var: Variance
