# Supplementary material for: DECONbench: a benchmarking platform dedicated to deconvolution methods for tumor heterogeneity quantification
Source: BMC Bioinformatics. 2021 Oct 2;22:473. doi: 10.1186/s12859-021-04381-4 (PMC8487526; doi:10.1186/s12859-021-04381-4)
Supplement: Supplementary file 1 — Additional file 1: Figure S1: DECONbench benchmark of OLS and RLR methods: an example of graphical outputs of new contributions to the benchmark. Source code. [file 12859_2021_4381_MOESM1_ESM.docx]

**Additional file 1**


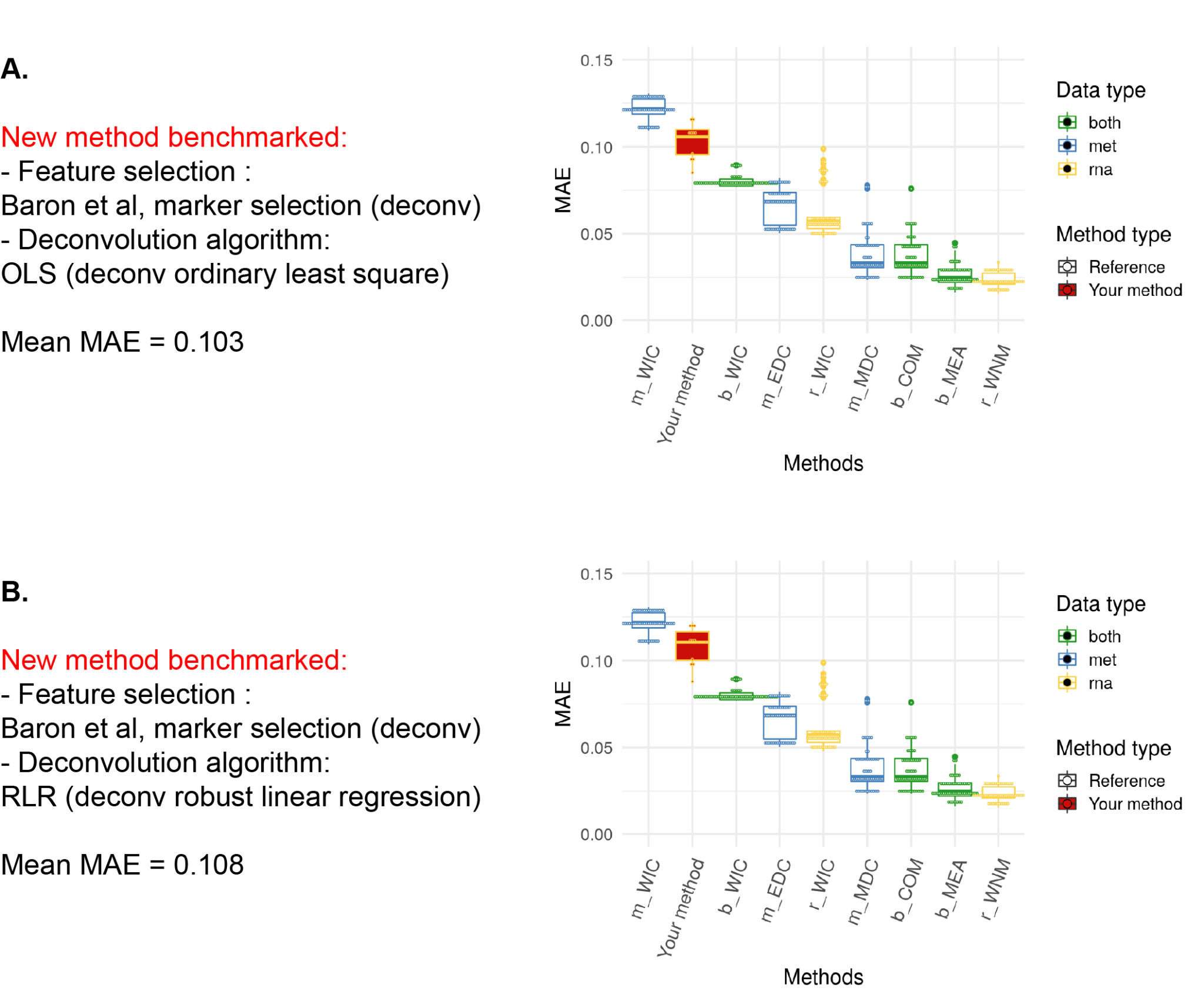


**Figure S1**: DECONbench benchmark of OLS and RLR methods: an example of graphical outputs of new contributions to the benchmark. For each novel submission, a graphical summary of the method performances is generated by the DECONbench platform, comparing performances of the participant’s method (“Your method”, in red), with the baseline methods. **A**. Boxplot of the Mean Absolute Error (MAE) of the estimation of the A matrices obtained by Ordinary Least Square deconvolution method (“Your method” in red), (OLS, from deconv pipeline analysis by Avila Cobos et al). **B**. Boxplot of the Mean Absolute Error (MAE) of the estimation of the A matrices obtained by Robust Linear Regression deconvolution method (“Your method”, in red), (RLR, from deconv pipeline analysis by Avila Cobos et al.).

**SOURCE CODE**

All codes used to generate the **supplementary figure 1** are greatly inspired by:

Avila Cobos, F., Alquicira-Hernandez, J., Powell, J.E. *et al.* Benchmarking of cell type deconvolution pipelines for transcriptomics data. *Nat Commun* 11, 5650 (2020). <https://doi.org/10.1038/s41467-020-19015-1>

and corresponding source code available at: **https://github.com/favilaco/deconv_benchmark**

‘Baron_deconv_markers.rds and ‘baron_deconv_refC.rds’ were generated using *deconv_benchmark Generator function* on Baron et al dataset.

(Baron:<https://www.ncbi.nlm.nih.gov/geo/query/acc.cgi?acc=GSE84133> )

DECONbench “OLS program” and “RLR program” are written below.

##################

### OLS program ###

##################

program <-

function(D_met = NULL, D_rna = NULL, k = 5) {

##

## YOUR CODE BEGINS HERE

##

#Loading the marker and the cell-type reference

marker_distrib = readRDS(paste0(submission_program ,"/baron_deconv_markers.rds"))

C = readRDS(paste0(submission_program ,"/baron_deconv_refC.rds"))

T = as.matrix(D_rna)

#Filtering C and T matrices

C = C[rownames(C) %in% marker_distrib$gene,]

T = T[rownames(T) %in% marker_distrib$gene,]

keep = intersect(rownames(C),rownames(T))

C = C[keep,]

T = T[keep,]

#Running deconvolution

RESULTS = apply(T,2,function(x) lm(x ~ as.matrix(C))$coefficients[-1])

RESULTS = apply(RESULTS,2,function(x) ifelse(x < 0, 0, x)) #explicit non-negativity constraint

RESULTS = apply(RESULTS,2,function(x) x/sum(x)) #explicit STO constraint

rownames(RESULTS) <- unlist(lapply(strsplit(rownames(RESULTS),")"),function(x) x[2]))

return(RESULTS)

##

## YOUR CODE ENDS HERE

##

}

dataType <-

"rna"

##################

### RLR program ###

##################

program <-

function(D_met = NULL, D_rna = NULL, k = 5) {

##

## YOUR CODE BEGINS HERE

##

#Loading the marker and the cell-type reference

marker_distrib = readRDS(paste0(submission_program ,"/baron_deconv_markers.rds"))

C = readRDS(paste0(submission_program ,"/baron_deconv_refC.rds"))

T = as.matrix(D_rna)

#Filtering C and T matrices

C = C[rownames(C) %in% marker_distrib$gene,]

T = T[rownames(T) %in% marker_distrib$gene,]

keep = intersect(rownames(C),rownames(T))

C = C[keep,]

T = T[keep,]

#Running deconvolution

require(MASS)

RESULTS = do.call(cbind.data.frame,lapply(apply(T,2,function(x) MASS::rlm(x ~ as.matrix(C), maxit=100)), function(y) y$coefficients[-1]))

RESULTS = apply(RESULTS,2,function(x) ifelse(x < 0, 0, x)) #explicit non-negativity constraint

RESULTS = apply(RESULTS,2,function(x) x/sum(x)) #explicit STO constraint

rownames(RESULTS) <- unlist(lapply(strsplit(rownames(RESULTS),")"),function(x) x[2]))

return(RESULTS)

##

## YOUR CODE ENDS HERE

##

}

dataType <-

"rna"
